# Meta‐analysis of test accuracy studies using imputation for partial reporting of multiple thresholds

**DOI:** 10.1002/jrsm.1276

**Published:** 2017-11-22

**Authors:** J. Ensor, J.J. Deeks, E.C. Martin, R.D. Riley

**Affiliations:** ^1^ Centre for Prognosis Research, Research Institute for Primary Care and Health Sciences Keele University Newcastle UK; ^2^ Institute of Applied Health Research, Public Health Building University of Birmingham Birmingham UK; ^3^ Manchester Pharmacy School The University of Manchester Manchester UK

**Keywords:** diagnostic test accuracy, imputation, meta‐analysis, multiple thresholds, publication bias

## Abstract

**Introduction:**

For tests reporting continuous results, primary studies usually provide test performance at multiple but often different thresholds. This creates missing data when performing a meta‐analysis at each threshold. A standard meta‐analysis (no imputation [NI]) ignores such missing data. A single imputation (SI) approach was recently proposed to recover missing threshold results. Here, we propose a new method that performs multiple imputation of the missing threshold results using discrete combinations (MIDC).

**Methods:**

The new MIDC method imputes missing threshold results by randomly selecting from the set of all possible discrete combinations which lie between the results for 2 known bounding thresholds. Imputed and observed results are then synthesised at each threshold. This is repeated multiple times, and the multiple pooled results at each threshold are combined using Rubin's rules to give final estimates. We compared the NI, SI, and MIDC approaches via simulation.

**Results:**

Both imputation methods outperform the NI method in simulations. There was generally little difference in the SI and MIDC methods, but the latter was noticeably better in terms of estimating the between‐study variances and generally gave better coverage, due to slightly larger standard errors of pooled estimates. Given selective reporting of thresholds, the imputation methods also reduced bias in the summary receiver operating characteristic curve. Simulations demonstrate the imputation methods rely on an equal threshold spacing assumption. A real example is presented.

**Conclusions:**

The SI and, in particular, MIDC methods can be used to examine the impact of missing threshold results in meta‐analysis of test accuracy studies.

## 1 INTRODUCTION

Primary studies that evaluate the diagnostic accuracy of a test that reports continuous results commonly report sensitivity and specificity values at multiple thresholds which define test positive and test negative patients. However, systematic reviews commonly observe heterogeneity in the thresholds chosen across studies, with not all thresholds reported in all studies. This inconsistent presentation of results for multiple thresholds creates a problem for researchers aiming to meta‐analyse test accuracy results across multiple studies, for example, to establish the best threshold for using the test in practise. Each threshold may have a different number of studies available, and there can be an abundance of information for some thresholds but a scarcity for others. For example, a recent study comparing aggregate and individual participant data (IPD) meta‐analyses of the Patient Health Questionnaire‐9 depression screening tool (PHQ‐9) identified 13 studies reporting on 13 different thresholds; 2 thresholds were only considered in 1 study, while the other 11 thresholds were considered in multiple studies, up to a maximum of 11 studies for any threshold.^1^ Therefore, none of the thresholds were reported in all studies. The issue of missing threshold information in diagnostic test accuracy meta‐analysis is highly prevalent, with many other examples in the literature.^2^, ^3^, ^4^, ^5^, ^6^, ^7^, ^8^

In such cases, it is common to meta‐analyse the results for each threshold separately, using the subset of 2‐by‐2 tables of test accuracy results available for each. Due to the lack of an established and validated alternative method, The Cochrane Handbook for Diagnostic Test Accuracy Reviews (chapter 10) suggests “Estimating summary sensitivity and specificity of the test for a common threshold, or at each of several different common thresholds” and notes that “Each study can contribute to one or more analyses depending on what thresholds it reports. Studies which do not report at any of the selected thresholds are excluded.”^9^ However, this approach excludes studies from a meta‐analysis if they do not report the threshold of interest, even if they did report results for other (often similar) threshold values. A further concern is selective reporting of threshold results in primary studies, where thresholds are more likely to be reported when they give large sensitivity and specificity results.^10^ This was highlighted in the aforementioned meta‐analysis of PHQ‐9 for depression screening,^1^ which showed that pooled sensitivity estimates were biased by between 5% and 15% in a meta‐analysis of published threshold results compared to using IPD. This potentially leads to over‐optimistic meta‐analysis results, biased toward larger pooled sensitivity and specificity results than the truth.

Many of these issues could be avoided if IPD were available, as tests reporting continuous results could then be analysed at a consistent threshold in all studies and therefore selective reporting could be avoided. However, here, our focus is on meta‐analysis of reported results. To address the issue of multiple thresholds per study, several methods have been proposed to synthesise results from multiple thresholds simultaneously, but most require a complete set of threshold results^11^, ^12^, ^13^ or require an approximate within‐study normality assumption on logit sensitivity and logit specificity estimates (with known within‐study variances), rather than modelling the 2‐by‐2 data directly.^14^, ^15^ Hamza et al^12^ proposed a multivariate random‐effect meta‐analysis approach which models the within‐study relationship between threshold value and test accuracy. An alternative survival model framework for meta‐analysing multiple thresholds was later proposed by Putter et al^13^ to counter convergence problems with the Hamza method. However, both these methods require all studies to report all thresholds of interest and therefore have limited applicability. Dukic and Gatsonis^11^ much earlier proposed a method for multiple thresholds, but it only allows a summary receiver operating characteristic (SROC) curve to be derived and does not give pooled estimates at each individual threshold of interest. Steinhauser et al recently proposed a novel method assuming either a normal or logistic distribution for the underlying test or biomarker and used a linear mixed model to allow estimation of an SROC curve and any desired threshold value (eg, Youden's index).^16^ The method requires the assumption of the distribution of the test results, which might not be known and may only be estimable if IPD are available.

Riley et al recently proposed a single imputation (SI) method to impute a 2‐by‐2 table for any missing threshold in a study that is bounded between 2 other available thresholds.^17^ A meta‐analysis can then be done at each threshold, with studies with imputed 2‐by‐2 tables synthesised with studies with known 2‐by‐2 tables. This was proposed as an exploratory method (sensitivity analysis) to examine the potential impact of the missing threshold results on meta‐analysis conclusions. The use of imputed results provides more information at each threshold allowing meta‐analysis results to be produced with more precision and, in the situation of selectively reported thresholds, potentially less bias. An empirical evaluation was previously undertaken to assess the performance of the SI approach, which showed promising results but no simulations were conducted to compare results to a known truth.^17^ Further, a concern is that the SI approach provides conservative standard errors for estimates of sensitivity and specificity, as it only includes a single imputed value, which ignores the uncertainty associated with it. In particular, the distance between a missing threshold and its nearest neighbour is ignored; in other words, the smaller the distance between 2 known thresholds, the more certain we should be about the imputed results for intermediate thresholds, but this is ignored in the SI approach. A final concern is conservative bias in the SI method, which is introduced through rounding down the number of patients in each cell of the imputed 2‐by‐2 tables (see Section 2.1).

Given these shortcomings, in this article, we propose a multiple imputation method based on discrete combinations of missing values (MIDC approach), to address the potential disadvantages for the SI approach. The proposed method imputes missing 2‐by‐2 tables between 2 known threshold results similar to the SI approach, but repeats this process on multiple occasions, each time using a randomly selected 2‐by‐2 table from the set of all possible discrete combinations of possible missing values. On each occasion, the imputed results are added to the meta‐analysis, and pooled results are estimated using standard methods for each threshold. These multiple sets of pooled estimates are then combined using Rubin's rules,^18^ as in standard multiple imputation applications,^18^, ^19^ to give overall pooled estimates of sensitivity and specificity for each threshold. In this way, the MIDC approach allows for the uncertainty in imputed threshold results and the distance between missing and known results.

A simulation study is used to evaluate the performance of the MIDC and SI approaches, in comparison with each other and the standard approach of only analysing observed data with no imputation (NI). Several scenarios are considered including varying the proportion of missing information, the missingness mechanism, the relationship between threshold value and test accuracy, and levels of heterogeneity between studies.

The remainder of the paper is structured as follows. Section 2 describes the methods of the SI and MIDC approaches in detail. Section 3 introduces the simulation study and describes the results. Section 4 illustrates the approaches using a real example, and Section 5 concludes with some discussion.

## 2 METHODS FOR SINGLE AND MULTIPLE IMPUTATION OF MISSING THRESHOLDS

We now briefly describe the SI method and then outline our new proposed MIDC method.

### 2.1 Single imputation of missing threshold results

The SI method follows a simple piecewise linear approach within each study separately, imputing a single value for any missing threshold results that are bounded between 2 known thresholds in logit ROC space.^17^ It assumes a unit increase in threshold value corresponds to a constant increase (or decrease) in logit specificity and logit sensitivity; such a linear relationship is often assumed in meta‐analyses that model the relationship across multiple thresholds,^12^, ^15^, ^16^ especially when the number of available thresholds is too small to examine non‐linearity.

Considering missing sensitivity results as an example, the following formula is used to impute the missing logit sensitivity threshold result (*y*_*ti*_) for threshold *t* in study *i*;

(1) $$y_{\mathrm{ti}}=y_{i\left(t-1\right)}+\left(\left(y_{i\left(t+1\right)}-y_{i\left(t-1\right)}\right)\times \left(\frac{\left(w_{\mathrm{it}}-w_{i\left(t-1\right)}\right)}{\left(w_{i\left(t+1\right)}-w_{i\left(t-1\right)}\right)}\right)\right),$$

where *y*_*i*(*t* − 1)_ is the observed logit sensitivity estimate for the nearest reported threshold below *t* and *y*_*i*(*t* + 1)_ is the observed logit sensitivity estimate for the nearest reported threshold above *t*. Weightings are given to each of the observed estimates, depending on how close their respective threshold values are to that of the missing threshold. If the missing threshold value is exactly in the middle of the 2 bounding thresholds, then the imputed *y*_*it*_ is simply the average of *y*_*i*(*t* − 1)_ and *y*_*i*(*t* + 1)_. Each pair of imputed logit sensitivity and logit specificity estimates is then converted back to a 2‐by‐2 table, using the known number of those diseased and non‐diseased in the study. It should be noted that the converted 2‐by‐2 table numbers can be noninteger values and that as such they often need to be rounded to produce a whole number, as otherwise statistical software (usually) will not recognise the data as binomial; here, we round them down to the nearest integer to be conservative when using the SI method.

Meta‐analysis is performed for each threshold separately, with any studies giving imputed 2‐by‐2 tables, synthesised along with studies providing an observed 2‐by‐2 table. A standard meta‐analysis model is used at each threshold separately, such as the bivariate random‐effects approach^20^, ^21^ that follows:

(2) $${\mathrm{TP}}_{\mathrm{ti}}\sim \text{Binomial}\left(D_{\mathrm{ti}},{\text{sensitivity}}_{\mathrm{ti}}\right)\text{logit}\left(\;{\text{sensitivity}}_{\mathrm{ti}}\right)=\beta_{t1}+u_{\mathrm{ti}1}{\mathrm{TN}}_{\mathrm{ti}}\sim \text{Binomial}\left({\mathrm{ND}}_{\mathrm{ti}},{\text{specificity}}_{\mathrm{ti}}\right)\text{logit}\left(\;{\text{specificity}}_{\mathrm{ti}}\right)=\beta_{t2}+u_{\mathrm{ti}2}\left(\begin{array}{c} u_{\mathrm{ti}1}\\ u_{\mathrm{ti}2} \end{array}\right)\sim N\left[\left(\begin{array}{c} 0\\ 0 \end{array}\right),\sum_{t}\right],\sum_{t}=\left(\begin{array}{cc} \tau_{t1}^{2} & \tau_{t1}\tau_{t2}\rho_{t12}\\ \tau_{t1}\tau_{t2}\rho_{t12} & \tau_{t2}^{2} \end{array}\right).$$

Here, *D_ti_* and *ND_ti_* give the number of diseased and non‐diseased in study *i*, *β*_*t*1_ gives the pooled logit sensitivity, and *β*_*t*2_ gives the pooled logit specificity at threshold *t*; *τ*_*t*1_ and *τ*_*t*2_ give the between‐study standard deviations at threshold *t*, and the off‐diagonals in ∑_*t*_ represent the between‐study covariance between logit sensitivity and logit specificity at threshold *t*. The analysis can be collapsed to a separate univariate analysis for both sensitivity and specificity, if the between‐study correlation *ρ_12_* is set to 0. Indeed, as the correlation is typically due to differences across studies in the threshold value, when analysing each threshold separately a correlation of 0 is quite plausible. In this simplified case, we are essentially fitting separate univariate models for sensitivity and specificity, thereby avoiding common convergence issues with the bivariate model.^22^ In practise, more complex meta‐analysis methods may be used post imputation, such as the joint modelling of all thresholds across all studies in a multivariate model, allowing for correlation across thresholds.^15^ In this study, the simpler approach of separate analyses at each threshold was used to facilitate comparison to a standard analysis approach in the simulation study, as this method is recommended (without imputation) in the Cochrane guidelines as standard.^9^ Given that separate analyses are performed at each threshold, throughout this article, between‐study heterogeneity is not due to different thresholds but instead due to other sources, for example, differences in population case‐mix or differences in the measurement method.

Riley et al^17^ suggested the SI approach as a simple exploratory method, to allow meta‐analysis results after imputation to be compared with those from a standard analysis with NI (ie, Equation (2) that simply excludes studies with missing threshold results).^9^, ^17^

### 2.2 Multiple imputation of missing threshold results based on discrete combinations

The schematic of the newly proposed MIDC approach is provided in Box 1. It follows 4 key steps, as now described.

#### 2.2.1 Step 1: identification and random selection of discrete combinations for imputing values for missing thresholds in each study separately

For each missing threshold bounded between 2 known thresholds, the MIDC method recognises that the missing sensitivity and specificity must lie within a rectangle formed between the 2 nearest known threshold results. Further, the missing number of true positives (TPs), false negatives (FN), true negatives (TN), and false positives (FP) at the missing threshold must be whole numbers of patients within this quadrilateral between those known for the neighbouring thresholds.

Table 1 shows an illustrative example of a study reporting the accuracy of a test with continuous results where there are 39 true diseased and 146 true non‐diseased patients. Assume that the thresholds of interest for meta‐analysis are 1, 2, 3, 4, and 5, but that this particular study only reports 2‐by‐2 tables for thresholds 1 and 5. Therefore, there are 3 missing thresholds (*r* = 3) to be imputed by the MIDC method, and it is clear that the missing TP must be ≤35 and ≥30; the missing FN must be ≤9 and ≥4; the missing TN must be ≤104 and ≥95; and finally, the missing FP must be ≤51 and ≥42. Therefore, there are 6 potential values (n = 6) for the TP in the missing thresholds (ie, 35, 34, 33, 32, 31, and 30) and 10 potential values for the TN in the missing thresholds (ie, 104, 103, … 96, and 95). It follows naturally that imputing TP also defines FN (or vice versa) given the total diseased, and imputing TN also defines FP (or vice versa), and therefore, we only need to consider imputing 2 cells in the missing 2‐by‐2 table, for example, TP and TN.

**Table 1:** Example data for a single study reporting a continuous test measured at a partial set of multiple thresholds of interest for meta‐analysis

Threshold	Missing	TP	FN	TN	FP
1	No	35	4	95	51
2	Yes	?	?	?	?
3	Yes	?	?	?	?
4	Yes	?	?	?	?
5	No	30	9	104	42

Abbreviations: FN, false negatives; FP, false positives; TN, true negatives; TP, true positives.

First, focus on imputing TP (and thus FN) for the 3 missing thresholds. There are 56 possible discrete combinations (of 3 values) for the TP to be imputed for the 3 missing thresholds, taking into account that the missing TP is bounded between 35 and 30 and that as threshold value increases the number of TPs must be equal to or less than the number of TPs at the previous threshold (see Table 2 for illustration). The MIDC method randomly selects one of these possible combinations, from the list of all combinations, assuming that all possible combinations are equally likely. In the same manner, a value for the missing TN (and FP) can be imputed for the same missing thresholds.

**Table 2:** First and last 5 of the 56 possible combinations of the imputed true positive (TP) values for thresholds 2, 3, and 4 in Table 1

First and Last 5 of 56 Combinations With Repetition for Imputed TP Values (n = 6, *r* = 3)			
Discrete combination n^o^	Threshold 2	Threshold 3	Threshold 4
1	35	35	35
2	35	35	34
3	35	35	33
4	35	35	32
5	35	35	31
52	32	30	30
53	31	31	31
54	31	31	30
55	31	30	30
56	30	30	30

The above describes a single imputation using the MIDC method in a single study for a particular set of missing thresholds. In each study separately, this approach can be used to impute TP, FN, TN, and FP for all missing but bounded thresholds. Note that, as in the SI method, in a particular study, there is no imputation for those thresholds that are not bounded (ie, those missing thresholds that fall above the largest reported threshold or below the smallest reported threshold).

#### 2.2.2 Step 2: meta‐analysis of imputed and observed 2‐by‐2 tables

In step 2, we now apply a meta‐analysis to each threshold separately, including the imputed and observed 2‐by‐2 tables available from the studies. For example, model 2 can be applied to produce summary logit sensitivity and logit specificity estimates and between‐study heterogeneity estimates.

#### 2.2.3 Step 3: generate multiply imputed datasets and multiple meta‐analysis results

Steps 1 and 2 are then repeated *M* times, leading to *M* meta‐analysis results (one for each cycle). As the imputation procedure generates new imputations in each cycle, the subsequent meta‐analysis results may also be different for each cycle.

#### 2.2.4 Step 4: combine across the multiple meta‐analysis results using Rubin's rule

The meta‐analysis estimates obtained from each of the *M* cycles are then combined using Rubin's rules, as commonly applied in traditional multiple imputation methods and detailed in full elsewhere.^18^ This provides final meta‐analysis estimates for each parameter of interest and its associated standard error (and thus 95% CI).

### 2.3 Potential advantages of the MIDC method over the SI method

By repeated random sampling from the set of all possible combinations of the missing 2‐by‐2 tables, the MIDC approach has 3 potential advantages over the SI method. Firstly, by considering multiply imputed datasets, it accounts for the uncertainty of the imputed 2‐by‐2 tables. Secondly, as it imputes the 2‐by‐2 tables directly, this ensures that all imputed values are whole numbers, which is not the case with the SI method. Thirdly, the method allows for the distance between known and missing threshold results, such that it is more likely that a missing threshold close to the known threshold will take a TP (or TN) value similar to the observed TP (or TN) value for the closest known threshold. This is illustrated for the current example in Table 3, where the probability of threshold 2 taking each of the 6 possible numbers of TPs is given; it is clear that it is most likely that a TP of 35 will be imputed for threshold 2, which is the observed TP value in the closest neighbouring threshold 1.

**Table 3:** Probability of each true positive (TP) value being imputed for missing threshold 2, which is bounded between 35 from threshold 1, and 30 from threshold 5

Possible TP Value	Probability of TP Value Being Imputed for Threshold 2	
	Fractional	Decimal
35	21/56	0.375
34	15/56	0.268
33	10/56	0.179
32	6/56	0.107
31	3/56	0.054
30	1/56	0.018

BOX 1. Discrete combinations (MIDC) approach schematic

Step 1 In each study separately,
  - identify all missing thresholds of interest that are bounded by 2 thresholds for which 2‐by‐2 tables are available.
  - for each set of missing thresholds contained within a bound, derive the set of all discrete combinations for the missing 2‐by‐2 tables.
  - randomly select a discrete combination from the set of all combinations, thereby imputing a single 2‐by‐2 table for all missing but bounded thresholds.
Step 2 For each threshold separately, apply a meta‐analysis (eg, model 2) to combine the imputed and observed 2‐by‐2 tables from all available studies, to produce 1 set of meta‐analysis results for each threshold.
Step 3 Repeat steps 1 and 2 a total of *M* times, to obtain *M* sets of meta‐analysis estimates for each threshold.
Step 4 Use Rubin's rules to combine the *M* meta‐analysis results for each threshold separately, to produce a final estimate and standard error for each parameter in the meta‐analysis model.

### 2.4 Software to implement the methods

Software was developed to implement the MIDC method within Stata and is available upon on request and will be submitted in due course for publication in the Stata journal. The software to implement the SI approach was previously developed and published elsewhere.^17^ The program follows the schematic laid out in Box 1 to implement the MIDC approach. In particular, each possible discrete combination has a unique combination number, which follows the pattern shown in Table 2. For each imputation, the discrete combination number is chosen randomly from a uniform distribution ranging from 1 to the total number of possible combinations. The corresponding combination is then identified, by exploiting the relationship between the unique combination number and cumulative sums of squares, which can be used to calculate the value at each missing threshold separately.

## 3 SIMULATION STUDY

A simulation study is now used to compare performance of the MIDC and SI methods with each other and also with the standard approach of ignoring missing thresholds which we refer to here onwards as NI.

### 3.1 Methods

Our simulation procedure was undertaken for each of a range of different scenarios. This followed a step‐by‐step process.

#### 3.1.1 Step 1: define the scenario

Table 4 shows the 15 different scenarios considered, covering different values for the amount of heterogeneity, the amount of missing data, the missingness mechanism, and the assumed threshold spacing. A simulation was conducted for each of the 15 scenarios. All simulations assumed that there were 10 studies available for the meta‐analysis, which is typical of the number available in practise.

**Table 4:** Simulation scenarios including base case and sensitivity scenarios

Scenarios	Studies	Prevalence	Tau	Missing %	Missing mechanism	Threshold spacing*
Base case						
1	10	10%	0	50	MCAR	Equal
2	10	10%	0.25	50	MCAR	Equal
3	10	10%	0.5	50	MCAR	Equal
Greater chance of missingness						
4	10	10%	0	70	MCAR	Equal
5	10	10%	0.25	70	MCAR	Equal
6	10	10%	0.5	70	MCAR	Equal
Missing not at random						
7	10	10%	0	50	MNAR	Equal
8	10	10%	0.25	50	MNAR	Equal
9	10	10%	0.5	50	MNAR	Equal
Unequal threshold spacing						
10	10	10%	0	50	MCAR	Unequal
11	10	10%	0.25	50	MCAR	Unequal
12	10	10%	0.5	50	MCAR	Unequal
Extreme unequal threshold spacing						
13	10	10%	0	50	MCAR	Extreme unequal
14	10	10%	0.25	50	MCAR	Extreme unequal
15	10	10%	0.5	50	MCAR	Extreme unequal

Abbreviations: MCAR, missing completely at random MNAR, missing not at random.

* Assumed threshold spacing.

#### 3.1.2 Step 2: generate the number of participants per study

For each meta‐analysis dataset of 10 studies, we randomly selected between 30 and 200 patients per study using a uniform (30,200) distribution.

#### 3.1.3 Step 3: generate the true disease status for each patient in each study

Prevalence of disease for all scenarios was set to 10% (see Table 4), with disease status in each meta‐analytic dataset being sampled from a Bernoulli (0.1) distribution, to reflect a typical disease prevalence. As an extension to the work, a prevalence of 50% was also investigated though not reported here (results discussed in Section 3.2.6 and available on request).

#### 3.1.4 Step 4: generate the true sensitivity and specificity values for each threshold in each study

The true sensitivity and specificity across thresholds of the test were simulated based on a real example diagnostic test accuracy meta‐analysis,^23^, ^24^ where the linear relationship between summary logit test accuracy and threshold value was available. In addition, varying levels of heterogeneity around the magnitude of the linear relationship were incorporated as defined by the scenarios given in Table 4. Alternatively simulated data could have been derived by assuming a distribution for the diseased and non‐diseased populations as has been done elsewhere^16^; further work could use this approach to assess multiple additional simulation scenarios assessing various distributions.

**Figure 1:**
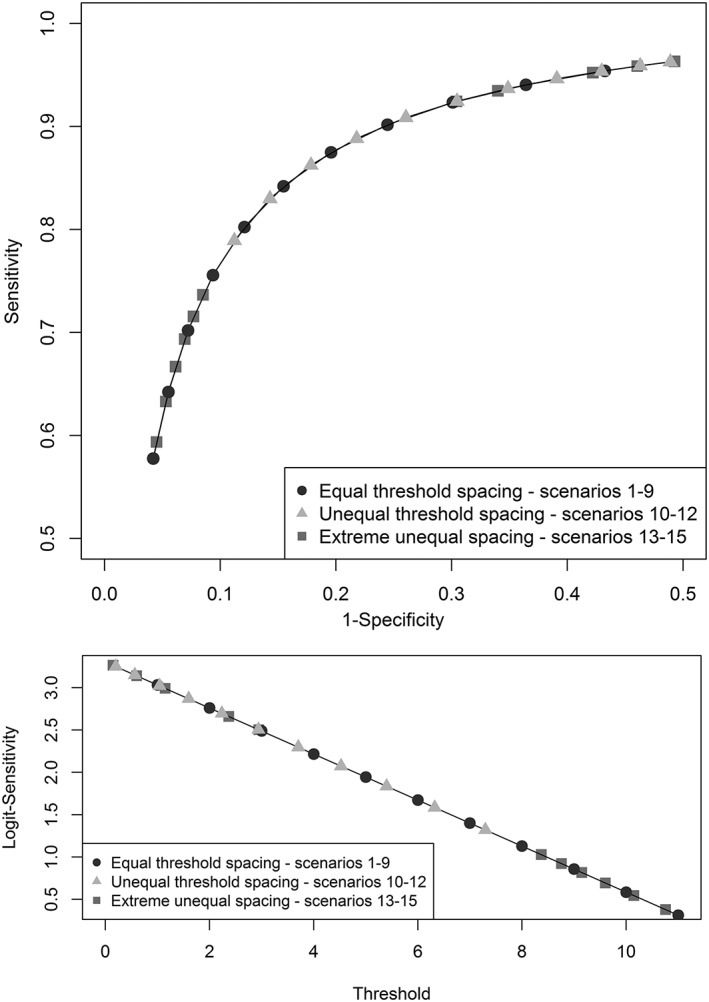
Mean summary receiver operating characteristic curves for the true test performance across thresholds (top panel), linearity of logit sensitivity across thresholds, and spacing of thresholds across scenarios (bottom panel)

The true sensitivity and specificity results at each threshold for each study were calculated using 2 linear models with either logit sensitivity or logit specificity as responses, and threshold value as an independent predictor (Equation (3)), this naturally induces a linear relationship between threshold value and logit sensitivity/specificity. The coefficients for the constant and threshold predictor for this assumed linear relationship were calculated based on those from the previous test accuracy meta‐analysis, as follows.^23^

(3) $$\text{\{\{True logit sensitivity in study}\;i\;\mathrm{at}\;a\;\text{threshold value}=\alpha_{1i}+\left(-0.2719091^{*}\;\text{threshold}\_\text{value}\right)\;\text{\{\{True logit specificity in study}\;i\;\mathrm{at}\;a\;\text{threshold value}=\alpha_{2i}+\left(0.2851818^{*}\;\text{threshold}\_\text{value}\right)\;{\{\{\alpha }_{1i}=N\left(3.304182,\tau^{2}\right),\;\alpha_{2i}=N\left(-0.0129091,\tau^{2}\right)$$

where τ^2^ defines the amount of between‐study heterogeneity, which was set to either be 0, 0.25 (moderate), or 0.5 (high) depending on the scenarios chosen (see Table 4). The mean SROC curve that this represents is shown in Figure 1 (top panel), and the assumed linear relationship is illustrated in Figure 1 (bottom panel) for logit specificity over threshold value.

For each study, the true intercepts were drawn randomly from the 2 normal distributions in Equation (3). This then produced 2 final equations to derive the true logit values, from which the true sensitivity (sens_*ti*_) and true specificity (spec_*ti*_) could be obtained for each threshold of interest (*t* = 1 to T) by back‐transforming from the logit scale.

Importantly, Equation (3) assumes a fixed slope for the threshold effect on logit sensitivity and logit specificity; we consider this a sensible starting point for examining the performance of the imputation methods. The fixed slope in Equation (3) is a common assumption made in diagnostic test accuracy meta‐analysis, though is often used because each study contributes only 1 threshold (2‐by‐2 table). In future research, this could be extended to allow the slope to vary between studies which may be a more realistic assumption.

Scenarios 1 to 9 assumed *equal* threshold spacing, but in scenarios 10 to 15 *unequal* threshold spacing was assumed (see Figure 1), with scenarios 13 to 15 investigating a more extreme unequal threshold spacing.

We chose 11 thresholds for all scenarios to be consistent, and this was based on the real example^23^ used to inform the parameters in Equation (3). The numerical values of the continuous test corresponding to the 11 thresholds were held constant across all studies within each scenario. The chosen threshold values were integer values from 1 to 11 for simulation scenarios 1 to 9; for scenarios 10 to 12, a transformation of these threshold values was used to induce unequal spacing, using Equation (4) as follows:

(4) $$\text{Threshold value for scenarios}\;10\;-\;12=\frac{{\left(\text{Threshold value scenarios}\;1-\;9\right)}^{1.5}}{5}.$$

Scenarios 13 to 15 were introduced as an extreme scenario to examine the effect of missing thresholds in the centre of the ROC curve. Here, we consider the extremely unequal spacing of thresholds as a combination of “bunching” of similar thresholds (at either end of the continuous measurement scale) and of “large gaps” between thresholds in the centre of the continuous scale (see Figure 1). In real applications, bunching of thresholds may be observed, for example, in meta‐analyses when a set of studies have used a very similar threshold but with differences in the last digit or decimal place. Large gaps may occur, for example, between thresholds chosen to optimise a test for rule out purposes (where a low threshold would be evaluated) or rule in purposes (where a high threshold would be evaluated). Threshold values were hand selected to create this effect for scenarios 13 to 15, with thresholds at 0.15, 0.6, 1.15, 2.37, 2.95, 8.37, 8.76, 9.15, 9.6, 10.15, and 10.76 (see Figure 1). It should be noted that scenarios 13 to 15 examine the effect of missing thresholds in the centre of the ROC curve, though a similar issue could occur elsewhere on the ROC curve.

#### 3.1.5 Step 5: generate the observed number of TP, TN, FP, and FN at each threshold

For each study separately, we next generated an observed 2‐by‐2 table for each threshold. To do this, we used the multinomial distribution expressed as a series of conditional binomial distributions.^12^ Firstly, to calculate the number of TPs above threshold 1 (the lowest threshold, *t* = 1), we randomly sampled *TP*_1*i*_ from a binomial distribution with *D_i_* = the total number of diseased in study *i*, and sens_1*i*_ = estimated sensitivity for threshold 1 in study *i*.

(5) $${\mathrm{TP}}_{1i}\;\sim \;\text{Binomial}\left(D_{i},{\text{sens}}_{1i}\right)$$

And thus, FN_1*i*_ = *D_i_* – TP_1*i*_.

For subsequent thresholds, the TP_*ti*_ were derived using a conditional binomial distribution. For example, we generated the number of TPs above threshold *2* out of the subset of patients who were positive above threshold 1, by calculating *TP*_1*i*_ minus a random sample from a $\;\text{Binomial}\left({\mathrm{TP}}_{1i},\frac{{\text{sens}}_{2i}}{\;{\text{sens}}_{1i}}\right)$ distribution. In this way, each successive TP_ti_ accounted for the previous TP_(*t* − 1)*i*_ value.

TN_*ti*_ and FP_*ti*_ for the non‐diseased population were generated in a similar manner for each threshold.

#### 3.1.6 Step 6: Create missing results for some thresholds

Step 5 produced complete data (ie, a 2‐by‐2 table) for each threshold in each study. To create missing data, the 2‐by‐2 tables in each study were removed using either a missing completely at random or, for scenarios 7 to 9, a missing not at random mechanism. For the missing completely at random scenarios, each 2‐by‐2 table was given a percentage probability of being missing according to the percentage missing data dictated by the scenarios setting (see Table 4). For the missing not at random scenarios, 2‐by‐2 tables could only be missing where the observed Youden's index was <0.7 (where Youden's index = sensitivity + specificity – 1 and therefore provides an overall measure of test performance). This reflects a situation where test performance is worse than expected at this threshold, and so it is vulnerable to publication bias and non‐reporting in the study publication. Such thresholds were given a 50% chance of being missing in scenarios 7 to 9. Other thresholds where Youden's index was at least 0.7 were always assumed to be available.

#### 3.1.7 Step 7: apply meta‐analysis to each simulated dataset using NI, SI, or MIDC methods

Steps 1 to 6 were applied to obtain 1000 meta‐analysis datasets in each scenario. For each dataset, the NI, SI, and MIDC methods were applied separately to produce 1000 meta‐analysis results for each type of approach. For the MIDC method, 5 imputation datasets were performed before Rubin's rules was applied (giving 1 meta‐analysis results dataset), 5 was selected to reduce the computation time of the overall simulation study. Meta‐analysis model 2 was used to synthesise the available results for each threshold separately but with the between‐study correlation set to 0 to avoid the common computation issues associated with this parameter.^22^, ^25^ The model was fitted via maximum likelihood estimation using Gauss‐Hermite quadrature with quadrature points equal to the number of studies in the meta‐analysis (up to a maximum of 5 quadrature points).

The performance of the 3 methods in each scenario was summarised and compared in terms of the bias in the mean of the pooled (logit) sensitivity and (logit) specificity estimates; the mean of the standard errors of the pooled results; the mean bias in the estimate of tau, and the percentage coverage of the 95% confidence intervals (CIs) for the pooled sensitivity and specificity. The 95% CIs were derived on the logit scale using the pooled estimates combined from all imputation datasets using Rubin's rules, and then back‐transformed.

### 3.2 Results

#### 3.2.1 Base case settings (scenarios 1 to 3)

The 3 base‐case scenarios each involved a 10% prevalence, 50% of thresholds missing completely at random, and equal threshold spacing, but varied according to the magnitude of between‐study heterogeneity. For each scenario, mean pooled estimates from the 1000 simulations from each of the NI, SI, and MIDC approaches were plotted at each threshold in ROC space and compared with the true ROC curve (Figure S1). When the heterogeneity was zero or moderate, there was very little bias at all thresholds, for either the NI method or the imputation methods. However, there was slight bias when the heterogeneity was large for all methods (Scenario 3), with pooled sensitivity and specificity underestimated across the thresholds. The bias was worst for the SI method, and the MIDC and NI methods performed similarly (Figure S1).

Coverage of 95% CIs was similar across the methods when there was no between‐study heterogeneity in scenario 1 (coverage ranged from 92% to 96%), with the NI method performing at least as well as the SI and MIDC methods (Figure S2). At moderate heterogeneity (scenario 2), the 2 imputation approaches had improved coverage over the NI approach (ie, closer to 95%) at most thresholds for specificity, while SI and MIDC performed similarly (Figure S2). For example, at a threshold of 5, the coverage for specificity was 89.3%, 92.1%, and 92.8% for the NI, SI, and MIDC approaches, respectively. The improvement in coverage by using the MIDC or SI approaches rather than NI was even more pronounced at a high level of between study heterogeneity (Figure S2), with MIDC performing best. For example, in scenario 3 with a threshold of 5, the coverage for specificity was 86.5%, 89%, and 90.6% for NI, SI, and MIDC approaches, respectively.

The improvement in coverage by using the imputation methods is most likely due to the estimate of between‐study heterogeneity being substantially improved by including more studies at each threshold after imputation (Figure S3). Maximum likelihood estimates of variances are known to be downwardly biased in small samples, and thus increasing the sample size via imputation ensures an improvement. Though downward bias in tau ( $\hat{\tau }$) remains for both MIDC and SI, it is far smaller than the bias for NI, and the MIDC method consistently provides the least biased estimates of tau ( $\hat{\tau }$) across the thresholds (Figure S3).

Despite the larger estimates of between‐study variance ( $\hat{\tau }$) after imputation, the incorporation of additional results via imputation substantially improves the precision of pooled estimates in the MIDC and SI approaches compared to NI (Figure S4). The gain in precision is largest in the central thresholds, where there is greater opportunity for imputation (ie, higher chance of a missing threshold falling between 2 known bounding thresholds); indeed, for these thresholds, the imputation approaches were usually able to recover data on all 10 studies in the meta‐analysis (see Supporting Information). Mean standard errors were almost identical for the SI and MIDC approaches, but with the MIDC method providing slightly inflated standard errors as it accounts for the uncertainty in imputations.

#### 3.2.2 Greater chance of missingness (scenarios 4 to 6)

In scenarios 4 to 6, the percentage of thresholds missing completely at random was increased to 70%. In terms of bias, the findings were similar to those in scenarios 1 to 3, with the pooled ROC curves showing very small bias for all the methods when the between study heterogeneity was large. Coverage and precision was again substantially improved by using the MIDC and SI approaches, even more so than for scenarios 1 to 3 due to the larger percentage of missing data. For example, at a threshold of 5 in scenario 6, the coverage was 78.9%, 88.6%, and 88.7% for the NI, SI, and MIDC methods, respectively. The 2 imputation methods were similar in terms of mean standard errors, which were reduced by up to 43% compared to the NI method.

#### 3.2.3 Missing not at random (scenarios 7 to 9)

**Figure 2:**
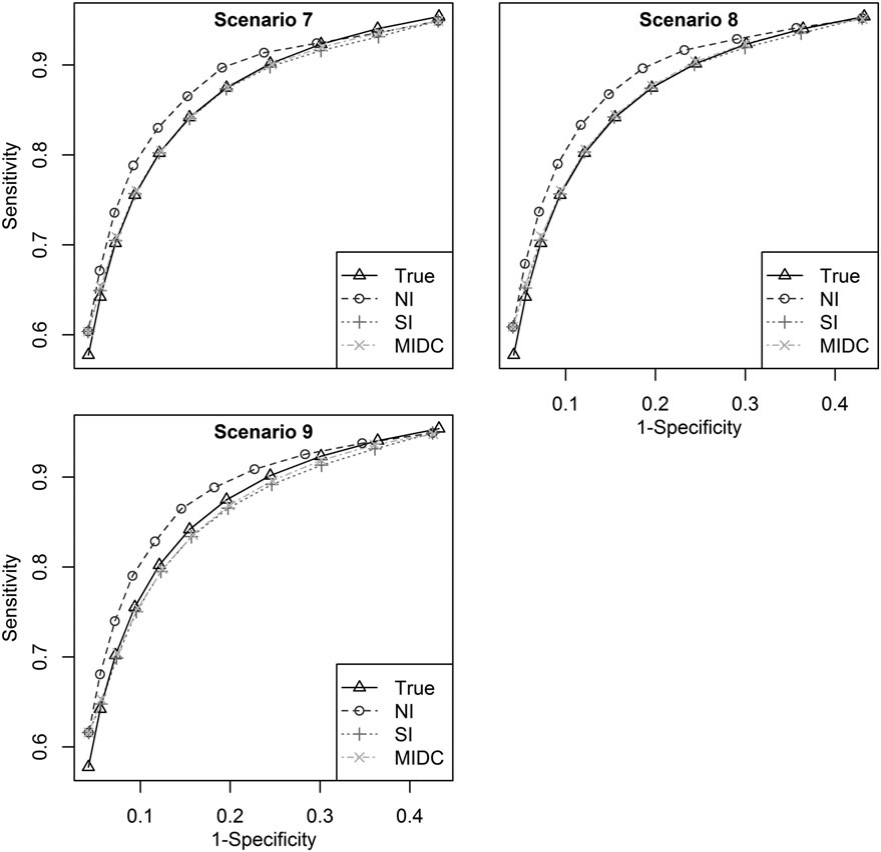
Summary receiver operating characteristic curves—scenarios 7 to 9

Under the missing not at random assumption in scenarios 7 to 9, a threshold was always present when the observed Youden's index was >0.7, but otherwise had a 50% chance of being missing akin to selective reporting bias. Figure 2 shows the SROC curves for each method from scenario 9 (high heterogeneity), and for the NI method, it reveals an upward bias (overestimation) of pooled sensitivity and specificity at each threshold when not accounting for the missing threshold data. There was also a similar upward bias for the NI method in scenarios 7 and 8, where there was lower heterogeneity. In contrast, the SI and MIDC methods reduce this bias through imputation and produce mean ROC curves that are close to the true SROC curve in each of scenarios 7 to 9.

Coverage of 95% CIs was again consistently better when using the imputation approaches than the NI approach. Differences between the SI and MIDC methods were generally small, though slightly better for the MIDC method when heterogeneity was large (Figure S5, scenario 9). Precision was also greatly increased when using either the SI or MIDC methods as previously noted.

#### 3.2.4 Unequal threshold spacing (scenarios 10 to 12)

**Figure 3:**
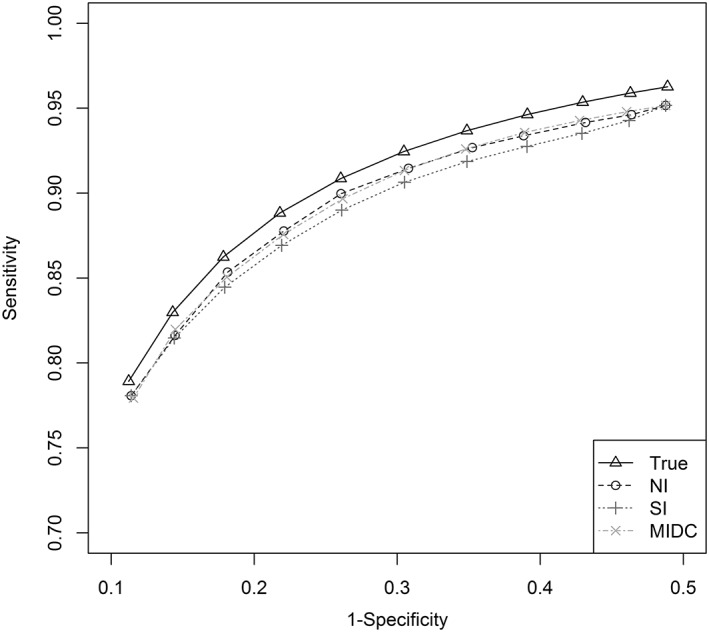
Summary receiver operating characteristic curves—scenario 12

All previous scenarios involved equal spacing of logit sensitivity and logit specificity across the range of thresholds. Scenarios 10 to 12 assess how the imputation methods perform given unequal threshold spacing along the ROC curve. Figure 3 presents the mean of the summary ROC curves for scenario 12 (high heterogeneity). The NI and MIDC methods showed some bias in pooled sensitivity and specificity estimates, while the SI method performed worst in terms of bias. The imputation methods again showed improved performance over the NI in terms of the estimated standard errors and between‐study variance, with the MIDC performing best. Coverage was generally best when using the MIDC method (Figure S6).

#### 3.2.5 Extreme threshold spacing (scenarios 13 to 15)

The impact of unequal threshold spacing was explicitly examined under more extreme unequal spacing situations (scenarios 13 to 15). These represented scenarios where thresholds were reported at either end of the ROC curve, but not reported in the central part of the ROC. This scenario may occur where studies are considering tests for rule in or rule out purposes as discussed in Section 3.1.

**Figure 4:**
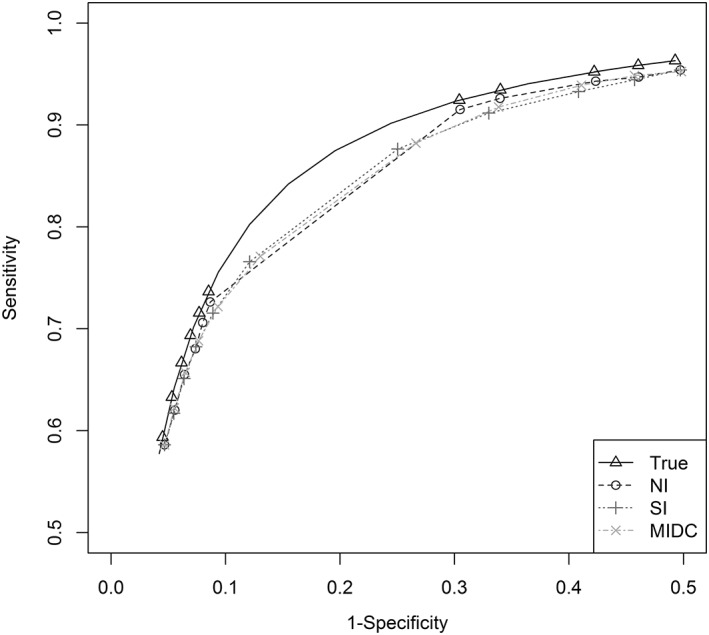
Summary receiver operating characteristic curves—scenario 15

Figure 4 presents the mean of the SROC curves for scenario 15 (high heterogeneity). Interestingly, the NI method showed little bias in sensitivity and specificity at the available thresholds; however, the 2 imputation methods performed poorly in terms of the central thresholds where averaging has occurred across the set of possible discrete combinations.

The coverage of 95% CIs appeared better when using either the SI or MIDC imputation approaches in the outer reported thresholds (Figure S7). However, the coverage of the imputation methods dropped substantially in the central thresholds. For example, in scenario 15 at a threshold of 5, the coverage for sensitivity was 96.4%, 63%, and 73.8% for the NI, SI, and MIDC, respectively, while at a threshold of 8, the coverage was 90.6%, 92.8%, and 93.8%, respectively. Precision of the pooled results was again improved using SI or MIDC compared to NI.

#### 3.2.6 Extension: prevalence of 50%

All simulations were repeated with a prevalence of 50%, rather than 10%. Simulations were also conducted with different numbers of studies, with 5 and 20 studies chosen to represent smaller and larger meta‐analysis datasets. Conclusions remained the same and results are available upon request. In particular, the NI approach generally performed worst, while the MIDC method performed generally best in terms of lower standard errors, coverage closest to 95%, and reduction in bias. It was observed that under the assumption of a 50% prevalence of disease, the coverage of 95% CIs for pooled sensitivity and specificity were similar, unlike at 10% prevalence, where the coverage of 95% CIs for pooled sensitivity was higher than those for pooled specificity (Figure S8). At 10% prevalence, the estimates of specificity will have far greater precision than sensitivity.

#### 3.2.7 Summary of findings

In general, across scenarios 1 to 9, where the equal spacing assumption was made, the simulations suggest that both the SI and MIDC methods perform better than the current standard NI method in terms of coverage and precision of summary sensitivity and specificity estimates, either when thresholds are missing completely at random or selectively reported according to Youden's index. This held for prevalences of either 10% or 50%. Improvements are due to the extra information arising from the imputed data, which also leads to improved estimation of the between‐study variances. Further, when there is selective reporting due to Youden's index, the findings suggest that the SI or MIDC methods can even reduce bias in the SROC curve, as well as improving coverage and precision. There is generally very little difference in the SI and MIDC methods, but the latter was noticeably better in terms of estimating the between‐study variances and generally gave better coverage, due to slightly larger standard errors of pooled estimates. However, when moderate unequal threshold spacing was assumed, the NI and MIDC methods performed better than the SI in terms of bias, with MIDC giving better estimation of standard errors and between‐study variances as before. However, under extreme unequal spacing the NI method performs best.

## 4 APPLIED EXAMPLE

We now illustrate the methods using an applied example based on a systematic review and meta‐analysis investigating the performance of protein/creatinine ratio (PCR) as a diagnostic test for the detection of significant proteinuria in patients with suspected pre‐eclampsia.^4^ This review is an ideal situation in which to apply the imputation methodology presented here. The review found 13 studies which reported various possible thresholds for PCR, with each study reporting on a different set of thresholds, making meta‐analysis difficult as discussed, due to small numbers of studies reporting on any 1 threshold. In total, there were 23 thresholds considered across all 13 studies, with 5 studies reporting only 1 threshold, and the largest meta‐analysis possible containing only 7 studies. The studies and 2‐by‐2 tables for each threshold are presented in online Supporting Information and are summarised elsewhere.^17^

The NI method reflects what happens with current meta‐analyses of test accuracy studies: Each threshold is analysed separately with NI. However, the use of the MIDC and SI methods here allows us to summarise not only the published evidence available for each threshold but also to use imputed data for the missing, unpublished evidence for bounded thresholds. As such, we are able to assess if the conclusions of the original meta‐analysis are robust (eg, to potential publication bias) for each threshold.

**Figure 5:**
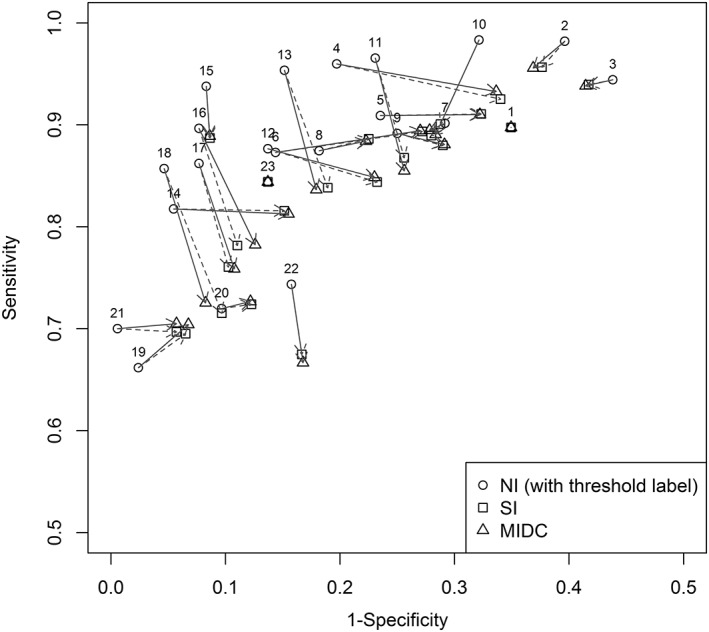
Protein/creatinine ratio data—shift in pooled estimates using single imputation (SI) and multiple imputation method based on discrete combinations of missing values (MIDC) vs no imputation (NI)

Figure 5 shows a dramatic change in pooled sensitivity and specificity (in ROC space) when using the MIDC method compared to the original NI approach. After imputation, it is clear that there is generally a shift in pooled estimates, both downward and to the right in ROC space. This indicates a decrease in sensitivity (downward shift) and specificity (shift right), as may be expected in the presence of publication bias or selective reporting, where weaker performing threshold results may not be reported. Therefore, the application of the MIDC method reveals that the original conclusions from the NI method are not robust, with pooled sensitivity and specificity estimates lower than estimated when ignoring missing data.

There are comparatively small differences between the MIDC and SI methods, with both indicating that pooled estimates shift in the same direction. The MIDC method shifts the pooled estimates by a slightly greater magnitude compared to the SI method, which is likely due to the MIDC method additionally accounting for the uncertainty in imputed threshold results and/or providing 2‐by‐2 tables that do not require rounding.

**Figure 6:**
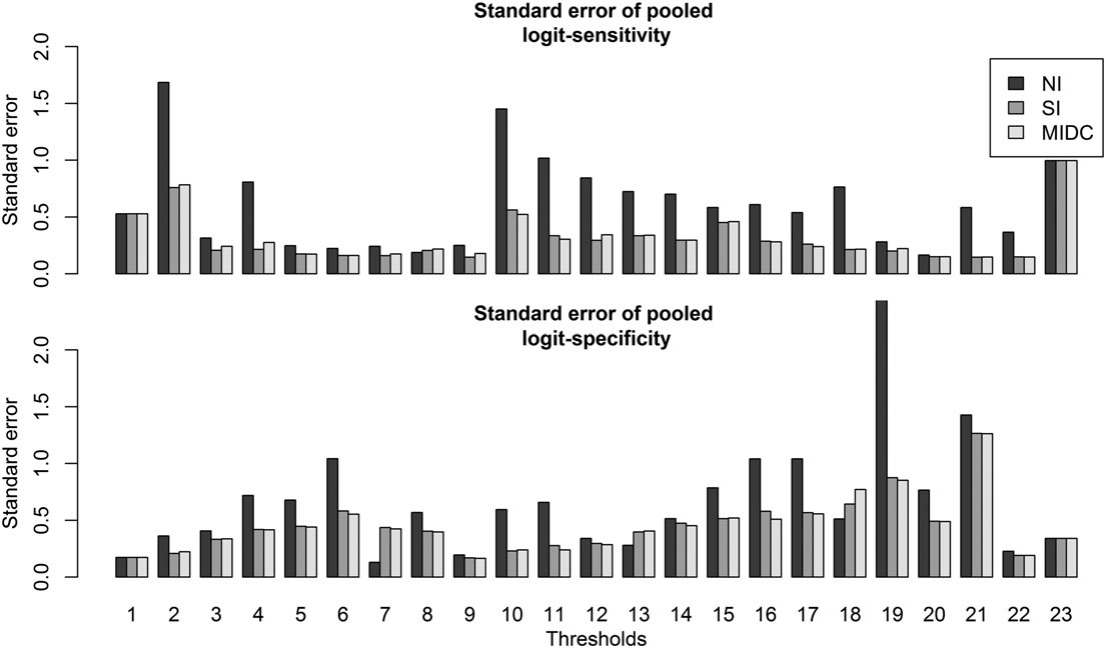
Standard error of pooled logit sensitivity (top panel), standard error of pooled logit specificity (bottom panel). MIDC, multiple imputation method based on discrete combinations of missing values; NI, no imputation; SI, single imputation

Figure 6 presents the standard errors of sensitivity and specificity at each threshold, showing the shift in standard error from the NI to MIDC method. Using the imputation approaches, we gained 54 additional threshold results for meta‐analysis, which reduced the standard errors of the pooled sensitivity and specificity at many thresholds (by as much as 70%, see Figure 6), also leading to substantially narrower CIs.

## 5 DISCUSSION

Often, test accuracy meta‐analyses suffer from having small and/or discrepant numbers of studies for each threshold of interest. In this paper, we proposed a new method to deal with this problem based on multiple imputation of all available discrete combinations of missing 2‐by‐2 tables. The results from our simulation study, across a wide‐range of scenarios, suggests that the previously proposed SI method and the new MIDC method help regain otherwise lost information, and generally improve performance of meta‐analysis results compared to the NI method. The SI and MIDC methods dramatically increase precision of pooled estimates of sensitivity and specificity at each threshold, as more data are added via imputation, which also often leads to a distinct improvement in coverage of 95% CIs compared to the standard NI method. This is especially evident when heterogeneity is large, as the SI and MIDC methods improve estimates of between‐study variance. Further, when thresholds are selectively missing due to a poor Youden's index, the findings suggest that the SI or MIDC methods reduce bias in the SROC curve compared to the NI method. In the situation of moderate unequal threshold spacing, the MIDC and NI methods perform better in terms of bias and coverage; and therefore, the threshold spacing assumption is important to the correct performance of the SI method. There is very little difference in most scenarios between the SI and MIDC methods; however, in a few scenarios, there was improved estimation of between‐study variances with the MIDC method and increased standard errors allowing for uncertainty in the imputation, sometimes leading to slightly better coverage. Results were found to be consistent at lower (10%) and higher (50%) levels of prevalence.

The MIDC method improves on the current SI method by allowing for uncertainty associated with the imputed threshold results and also uncertainty associated with the distance between the missing threshold and the nearest known threshold results. The MIDC method is more complex as it imputes missing threshold results multiple times and combines these results to give 1 pooled performance at each threshold; this inevitably increases computation time, but only slightly (typically 10 and 20 seconds for SI and MIDC, respectively). While this work focuses on the application to tests reporting continuous results, future research could extend these methods for use with tests reporting ordinal results. The software to implement the MIDC procedure in Stata is available upon request and will be submitted in due course for publication in the Stata journal.

There has been debate concerning the order in which meta‐analysis and Rubin's rules should be performed, as such the MIDC approach performs meta‐analysis on the imputed dataset and then applies Rubin's rules after meta‐analysis, as Rubin's theory and recent evidence suggests.^18^, ^26^

As with common multiple imputation methods, the number of MIDC datasets (or imputations) can be increased to reduce uncertainty further; due to the large computation time of the simulations, only 5 MIDC datasets were used throughout the simulation study. However, in reality sensitivity, analyses could be conducted in individual cases to decide on an appropriate number of MIDC datasets to reduce uncertainty as required. One possible recommendation could be to ensure *M* is set to be 100*p, where p is the proportion of missing studies for the threshold with the most missing data that can be imputed, to be consistent with guidance elsewhere.^19^, ^27^ Another limitation of our simulation study was the restriction to 10 studies. This was chosen to reduce the computation time required to conduct the simulations, and because it is typical of many meta‐analyses in the test accuracy field.

It is important to note that any meta‐analysis model may be used for synthesis following the use of either imputation approach. In this study, we chose to synthesise each threshold separately using separate univariate models for sensitivity and specificity (by setting the between‐study correlation to 0 in the bivariate model). While this is a simplifying assumption, it is one commonly used in most diagnostic test accuracy meta‐analyses in the current literature (eg, in Cochrane reviews). However, other more complex multivariate methods may be used post imputation to synthesise all thresholds across all studies simultaneously allowing for all correlations. Further research considering the use of such methods after our imputation strategy is needed.

There are limitations of the MIDC method, however. In situations of extreme unequal spacing, the MIDC and SI methods perform less well, and so, judgement is needed as to whether this is likely to be the case in particular examples. Therefore, a key issue is how we detect extreme unequal spacing and how we draw conclusions from the imputation methods in these cases; further research is needed to investigate these questions. One cause of this problem (illustrated in the extreme unequal spacing scenarios) is “bunching,” where we have a set of thresholds across studies of which some are very close together (for example, say that we had thresholds of a biomarker at 10, 20, 20.1, 20.15, 20.2, 20.25, 20.3, 30, 40, and 50). Where bunching occurs, 2 possible solutions could be (1) to group thresholds that are very close together or (2) to select a subset of thresholds for analysis, so that the thresholds are roughly equally spaced. The effects of different subsets of thresholds could then be investigated in sensitivity analyses.

Also, the SI method uses a linearity assumption in the change in logit sensitivity and logit specificity as the threshold value increases by 1 unit. Steinhauser et al assume a distributional shape for both the diseased and non‐diseased population, but assessing the true distribution of the test would require IPD (eg, for at least 1 study) across the range of the test, which will often not be available.^16^ Further work may aim to assess how suitable the linearity assumption is and to what extent deviation from this distribution matters for the SI approach. The multiple imputation approach for the MIDC method used discrete combinations, but an alternative approach may be to assume some distribution for the diagnostic test which would allow potential imputations to be drawn from some posterior distribution as in standard multiple imputation approaches; again, this would require the distribution of the test be known, and we feel this will often be unlikely and impractical for those researchers (often non‐statisticians) who are undertaking such meta‐analyses.

The biggest limitation of the imputation methods is that neither the MIDC or SI approaches constrain the ordering of threshold results, meaning that in real‐world examples, the imputed results may lead to imperfect SROC curves (as seen in the PCR example).^15^ The methods of Hamza et al and Riley et al constrain the threshold results to be ordered; however, these methods are limited by requiring either complete data or a normality assumption on the logit estimates within studies.^12^, ^15^ Further work may look to address this problem.

It is also important to note that the imputation methods proposed here can only be used with tests reporting continuous results, that is, reporting sensitivity and specificity at some numerical threshold. For example, the methods would not apply to tests defined solely in terms of “Normal,” “Possibly Abnormal,” and “Abnormal,” as this would form a categorical test and the threshold for each category may be subjective. The imputation methods assume an explicit threshold is defined and would apply if the categories “Normal,” “Possibly Abnormal,” and “Abnormal” were actually defined by some underlying numerical thresholds of a continuous measure.

In conclusion, we recommend the use of the MIDC method in practise as a sensitivity analysis. For example, researchers who retain the NI approach as their primary analysis method, could subsequently use the MIDC method to investigate the impact of missing threshold information on the summary sensitivity and specificity of the test. This could be particularly important in flagging that pooled test accuracy may be weaker than originally thought from the NI results, perhaps due to publication bias related issues. Similarly, if applicable, other methods for dealing with multiple thresholds might be considered.^11^, ^12^, ^13^, ^15^

## CONTRIBUTORS

JE is the guarantor. JD developed the concept of using discrete combinations for this problem. JE, JD, and RR developed the MIDC method. JE developed the code to implement the method, with contribution from EM toward the coding of discrete combinations. JE designed and conducted the simulation study, with input from all authors. JE wrote the first draft of the manuscript, and all authors contributed to subsequent revisions. All authors read and approved the final manuscript.

## CONFLICTS OF INTERESTS

The authors declare that they have no known competing interests.

## Supporting information

Data S1. Supporting Information

Figure S1 ROC curves compared to true estimates (base case scenarios 1–3)
Figure S2 Coverage of 95% confidence intervals (base case scenarios 1–3)
Figure S3 Estimate of tau for sensitivity for scenario 2 (tau = 0.25) and scenario 3 (tau = 0.5)
Figure S4 Standard errors (base case scenarios 2–3)
Figure S5 Coverage of 95% confidence intervals for MNAR scenario 9
Figure S6 Coverage of 95% confidence intervals for unequal threshold spacing scenario 12
Figure S7 Coverage of 95% confidence intervals for extreme unequal threshold spacing scenario 15
Figure S8 Coverage of 95% confidence intervals for MNAR scenario 9. Comparing simulations results at 10% prevalence (top figures) and 50% prevalence (bottom figures).
