# Supplementary material for: Meta‐analysis of test accuracy studies using imputation for partial reporting of multiple thresholds
Source: Res Synth Methods. 2017 Nov 22;9(1):100–15. doi: 10.1002/jrsm.1276 (PMC5873416; doi:10.1002/jrsm.1276)
Supplement: Supplementary file 1 — Data S1. Supporting Information [file JRSM-9-100-s001.pdf]

Supplementary figures

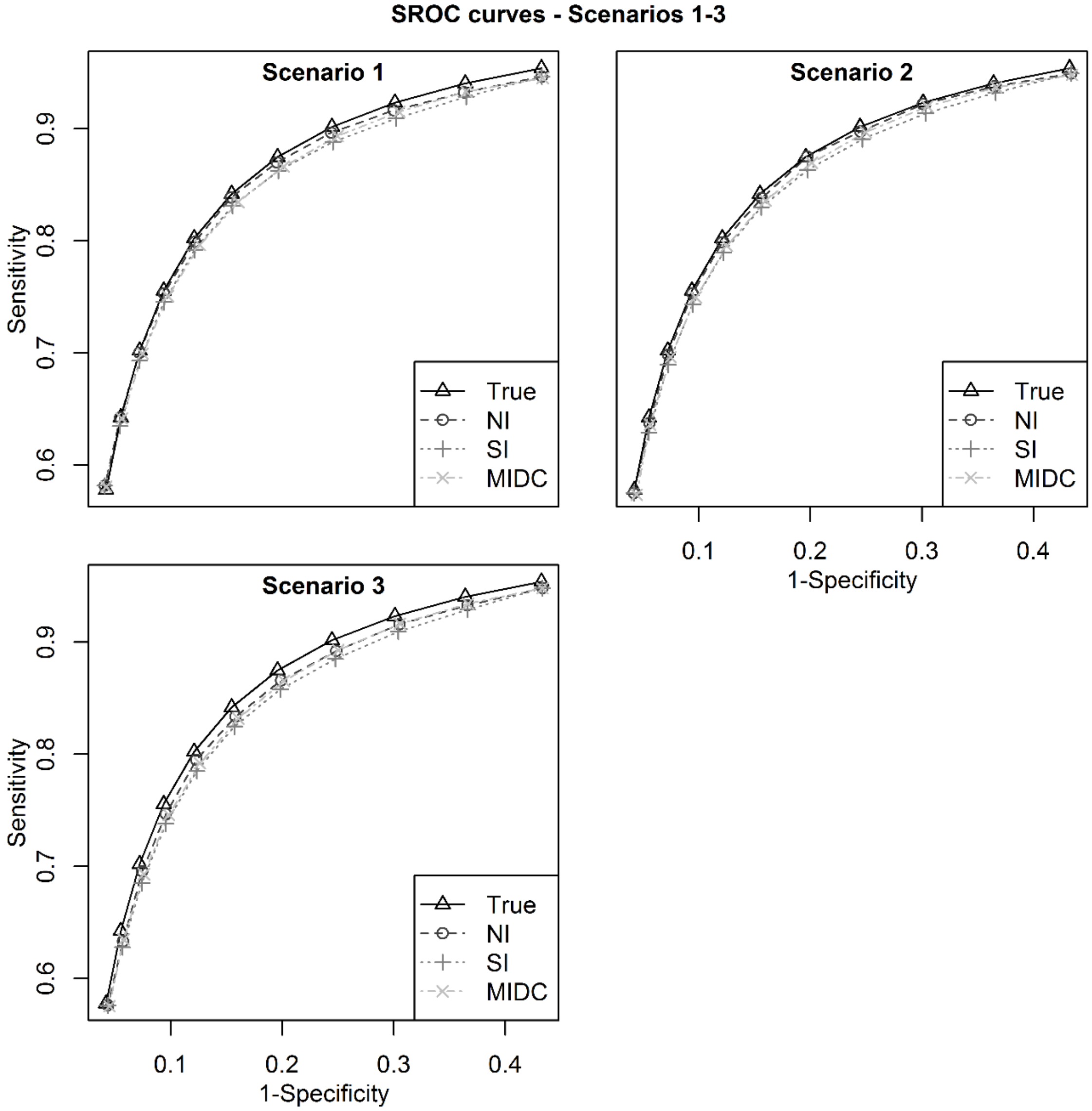

Figure S1 - ROC curves compared to true estimates (base case scenarios 1-3)

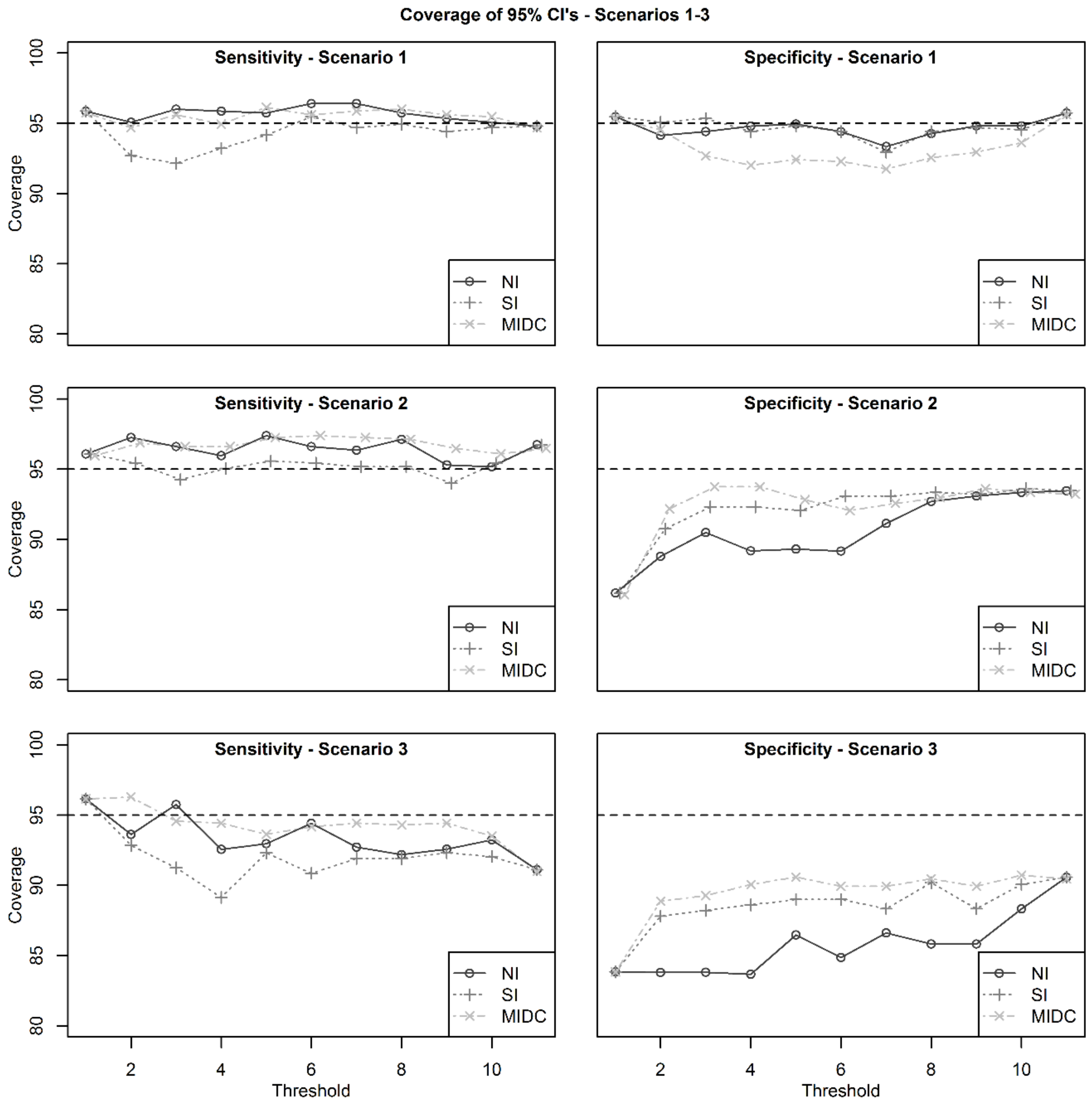

Figure S2 - Coverage of 95% confidence intervals (base case scenarios 1-3)

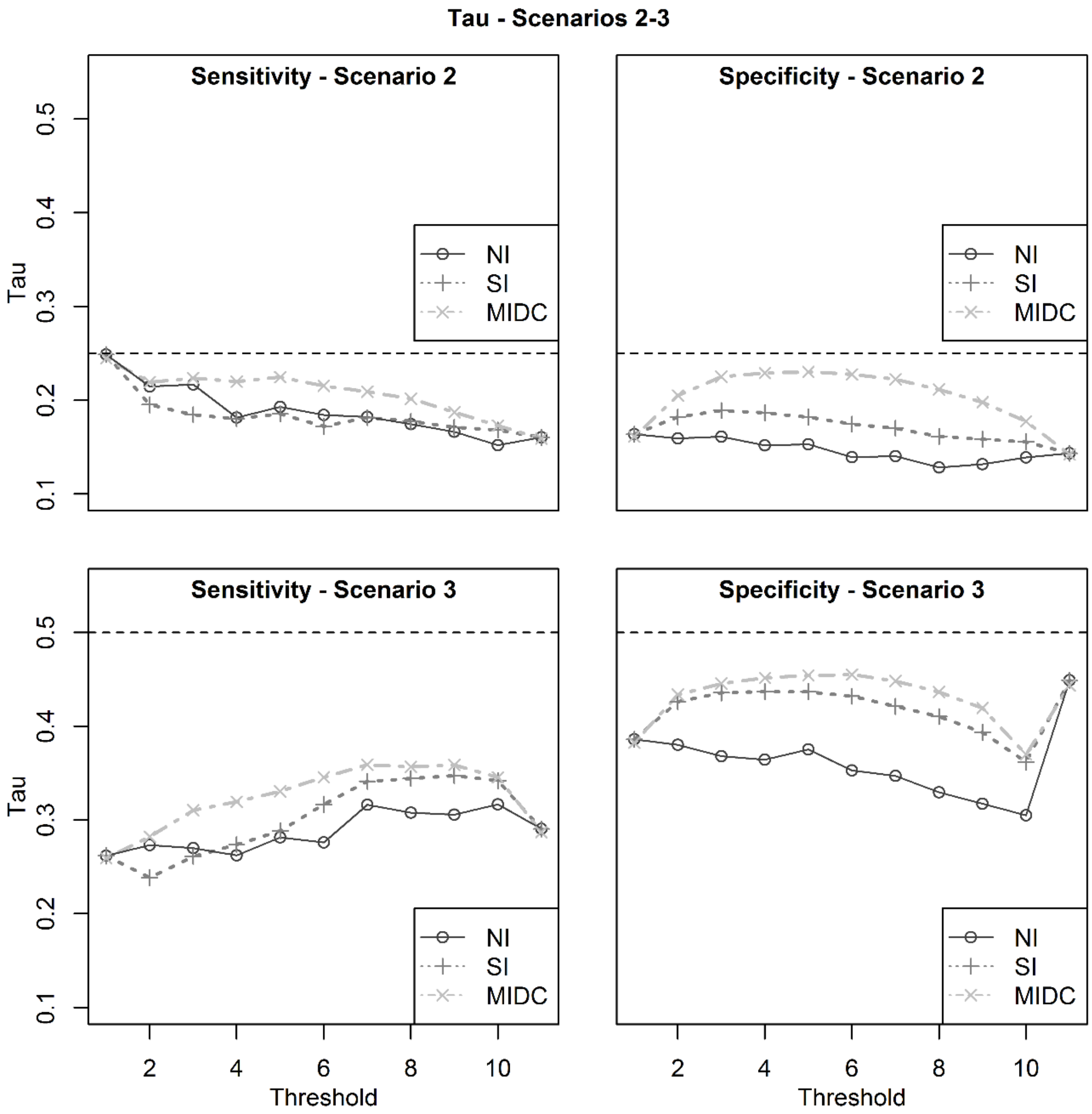

Figure S3 - Estimate of tau for sensitivity for scenario 2 (tau=0.25) and scenario 3 (tau=0.5)

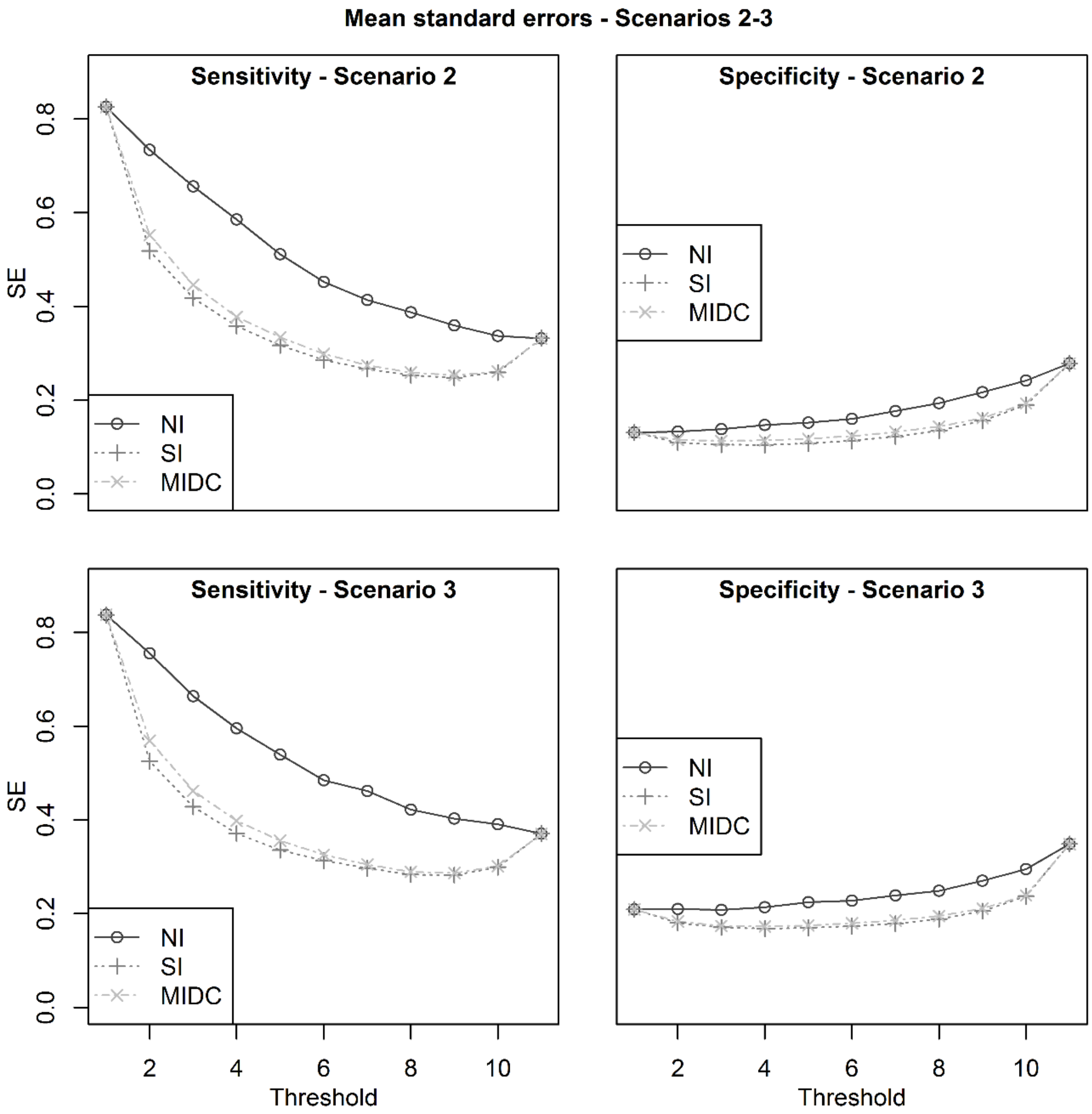

Figure S4 - Standard errors (base case scenarios 2-3)

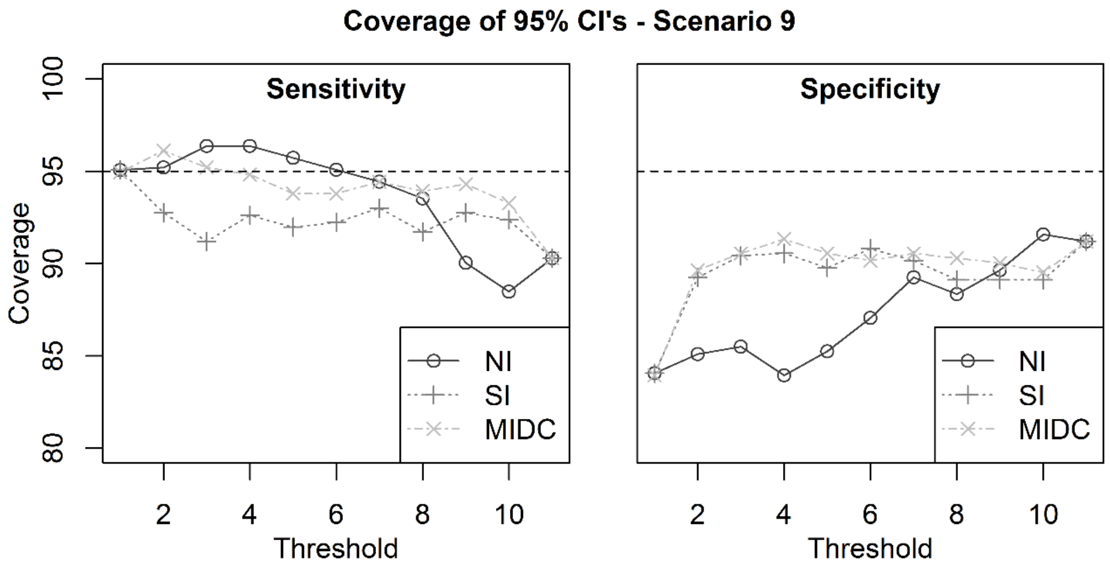

Figure S5 - Coverage of 95% confidence intervals for MNAR scenario 9

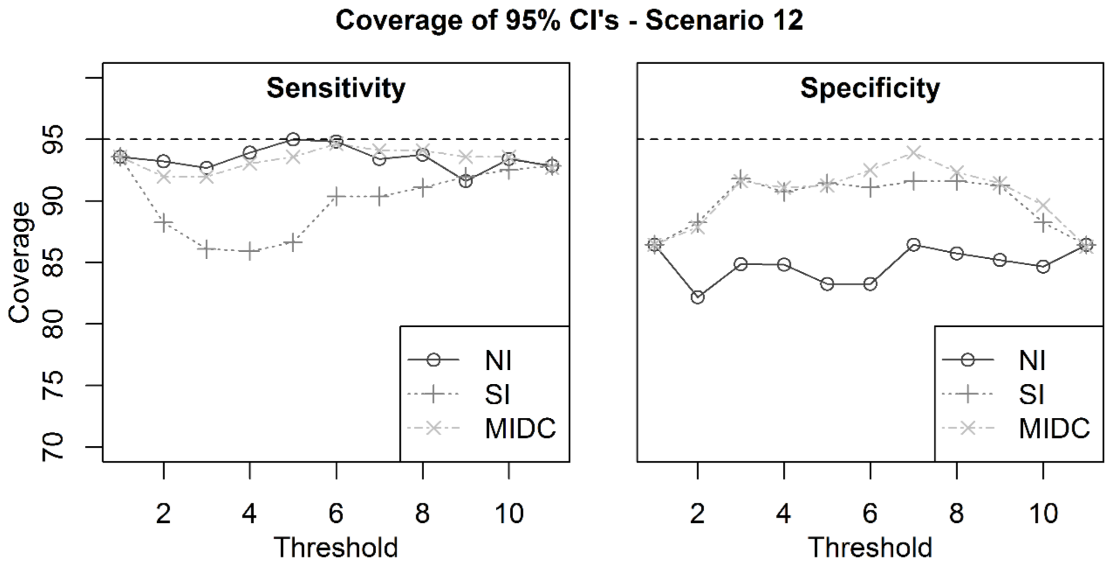

Figure S6 - Coverage of 95% confidence intervals for unequal threshold spacing scenario 12

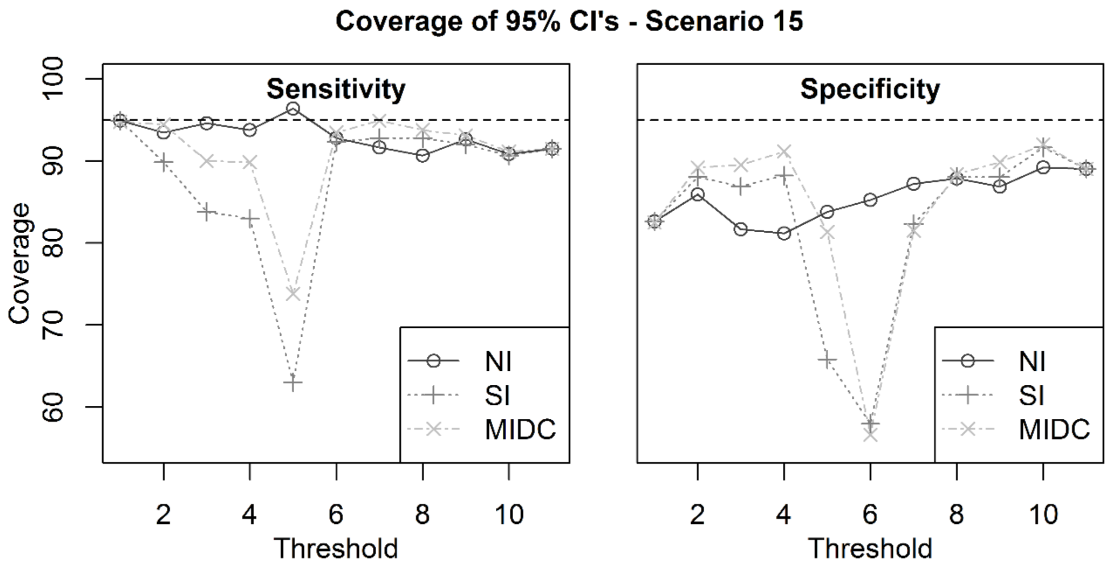

Figure S7 - Coverage of 95% confidence intervals for extreme unequal threshold spacing scenario 15

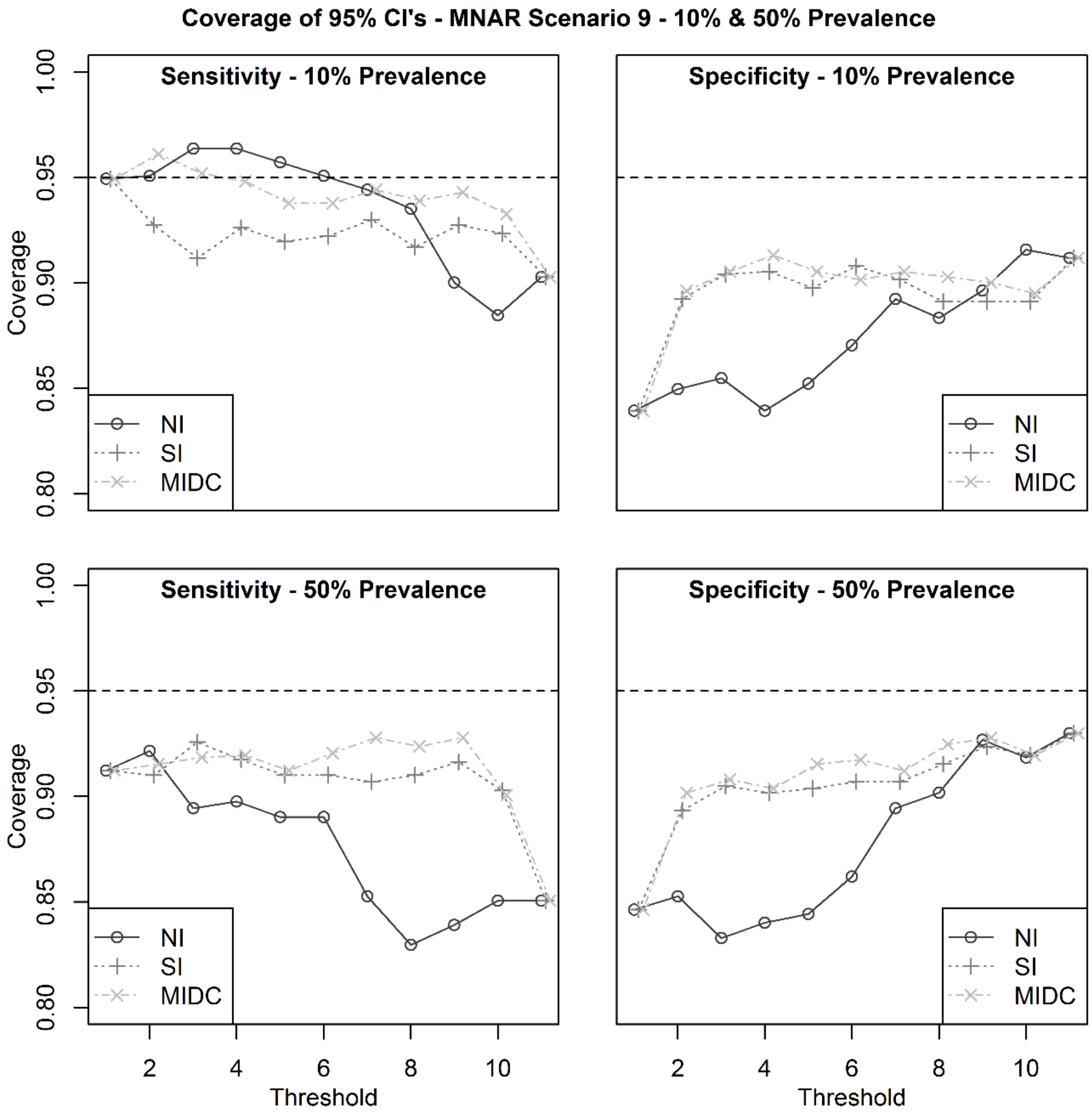

Figure S8 - Coverage of 95% confidence intervals for MNAR scenario 9. Comparing simulations results at 10% prevalence (top figures) and 50% prevalence (bottom figures).
